# Three Surgical Cases of Cecal Volvulus

**DOI:** 10.7759/cureus.72794

**Published:** 2024-10-31

**Authors:** Makoto Takahashi, Yuji Ando, Saki Kochi, Miyuki Toake, Hiromitsu Takahashi, Aya Kobari, Kumpei Honjo, Masaya Kawai, Kiichi Sugimoto, Kazuhiro Sakamoto

**Affiliations:** 1 Department of Coloproctological Surgery, Juntendo University, Tokyo, JPN

**Keywords:** cecal volvulus, cecopexy, computed tomography, emergency surgery, indocyanine green fluorescence imaging, mobile cecum

## Abstract

Cecal volvulus (CV) is a relatively rare disease; however, it often requires emergency surgery due to the low success rate of endoscopic treatment, in contrast to sigmoid volvulus. The mechanism of CV involves a mobile cecum at the base, triggered by factors such as constipation, high-fiber diets, laxative use, history of laparotomy or laparoscopic surgery, pregnancy, and prior colonoscopy, which twists the ileocecal region. Although CV is a benign disease, it can be fatal if treatment is delayed, so it is crucial to understand the pathophysiology and treatment. In this report, we describe three cases of CV surgery that were all treated within a relatively short period. The patient in case 1, who was a woman in her 70s on steroid therapy for collagen disease, had abdominal fullness. CV was diagnosed by computed tomography (CT), and emergency surgery was performed. Indocyanine green (ICG) fluorescence imaging was performed intraoperatively to confirm that blood flow in the ileocecal area was intact, after which a cecopexy was performed. Case 2 revealed abdominal fullness in a woman in her 50s undergoing depression treatment. The CT diagnosis of CV led to the performance of emergency surgery. The intraoperative findings revealed intestinal ischemia, leading to the performance of an ileocecal resection. Case 3 involved a man in his 70s, suffering from cardiac amyloidosis and heart failure (HF) and in a long-term bedridden state, who was experiencing abdominal pain. The patient was diagnosed with CV by CT, but due to his poor general condition, colonoscopic treatment was first attempted. The endoscopic findings revealed intestinal strangulation, so ileocecal resection was performed on the following day. The patients in the three CV cases were relatively elderly, and all had some comorbidities. CT was effective for diagnosing CV, and emergency surgeries were necessary. The use of ICG imaging and the performance of cecopexy were also important, depending on the patient's condition.

## Introduction

Cecal volvulus (CV) is a relatively rare disease, with an incidence of about 1% of intestinal obstructions and 15-30% of all cases of colonic volvulus [1]. CV is considered to occur in patients with an underlying mobile cecum and is triggered by factors such as chronic constipation, high-fiber diets, laxative use, history of laparotomy or laparoscopic surgery, pregnancy, and prior colonoscopy [2]. Compared to sigmoid volvulus, the success rate of endoscopic intervention for CV is low, at about 30%, and immediate surgery is recommended [2]. Ileocecal resection is the most common procedure, but cecopexy and cecostomy are also performed. Cecopexy may be performed in unstable, high-risk patients when there is no evidence of ischemia or perforation, but the high recurrence rate is problematic. Cecostomy is also performed in high-risk patients; however, the atrophic or edematous tissue makes seromuscular suture difficult, and postoperative complications such as leakage from the suture site, necrosis of the cecum, and fistula may occur. Considering these factors, ileocecal resection is considered the golden standard [3,4]. We experienced three surgical cases of CV in a relatively short period of time. Two patients underwent ileocecal resection, and one underwent cecopexy using indocyanine green (ICG) fluorescence imaging. Although CV is a relatively rare benign disease, understanding the diagnosis, treatment, and surgical techniques is crucial, and we present the three cases to enhance this understanding.

## Case presentation

Case 1

The patient was a woman in her 70s, height 153 cm and weight 35 kg, with a BMI of 15.1 kg/m^2^. A surgical incision scar from a uterine fibroid treated about 30 years ago was present in the midline of the lower abdomen. She was attending an outpatient department of internal medicine for mixed connective tissue disease and had been taking prednisolone 5 mg/day for over 20 years. In addition, she had coexisting interstitial pneumonia, hypertension (HT), diabetes mellitus (DM), hyperlipidemia, and glaucoma, and she had been stented for a thoracic aortic aneurysm several years earlier, taking antiplatelet drugs (Table 1).

**Table 1:** Characteristics of the three cases CT: computed tomography; CS: colonoscopy; ICG: indocyanine green; CV: cecal volvulus; MCTD: mixed connective tissue disease; IP: interstitial pneumonia; TAA: thoracic aortic aneurysm; HF: heart failure; CKD: chronic kidney disease.

Case	Age	Sex	Major backgrounds	Preoperative CT	Whirl sign	Preoperative CS	Preoperative diagnosis	Rotation	ICG	CV type	Procedure
1	70s	Female	MCTD, IP, TAA	(+)	(+)	(−)	Cecal volvulus	270°	(+)	Ⅱ	Partial colectomy + cecopexy
2	50s	Female	Depression	(+)	(+)	(−)	Cecal volvulus	270°	(−)	Ⅱ	Ileocecal resection
3	70s	Male	HF, CKD, bedridden	(+)	(+)	(+)	Cecal volvulus	360°	(−)	Ⅱ	Ileocecal resection

The patient visited our hospital with abdominal distention, abdominal pain, and vomiting. On arrival, she had marked abdominal distention and tenderness around the umbilicus. Her vital signs at the time of admission were blood pressure (BP) 128/68 mmHg, heart rate (HR) 72 beats per minute (bpm) and regular, and body temperature (BT) 36.4 °C. Blood tests showed an elevated inflammatory response with a white blood cell (WBC) count of 12,500/μL and a C-reactive protein (CRP) level of 6.62 mg/dL (Table 2).

**Table 2:** Laboratory results of the three cases WBC: white blood cell; Hb: hemoglobin; Plt: platelet; AST: aspartate aminotransferase; ALT: alanine aminotransferase; CK: creatine kinase; Alb: albumin; BUN: blood urea nitrogen; Cre: creatinine; CRP: C-reactive protein.

	Normal range	Case 1	Case 2	Case 3
WBC (/µL)	3,900–9,700	12,500	9,200	4,000
Hb (g/dL)	13.4–17.1	12.9	15.3	12.2
Plt (10^3^/μL)	15.3–34.6	28.9	25.4	6.4
AST (U/L)	5–37	12	16	29
ALT (U/L)	6–43	7	15	9
CK (U/L)	57–240	25	62	123
Alb (g/dL)	4.0–5.2	3.7	4.0	3.4
BUN (mg/dL)	9–21	22	13	14
Cre (mg/dL)	0.6–1.0	0.49	0.65	0.92
Na (mmol/L)	135–145	148	138	135
K (mmol/L)	3.5–5.0	3.9	3.7	3.6
Cl (mmol/L)	96–107	111	99	99
CRP (mg/dL)	0–0.29	6.62	12.11	4.64

**Figure 1:**
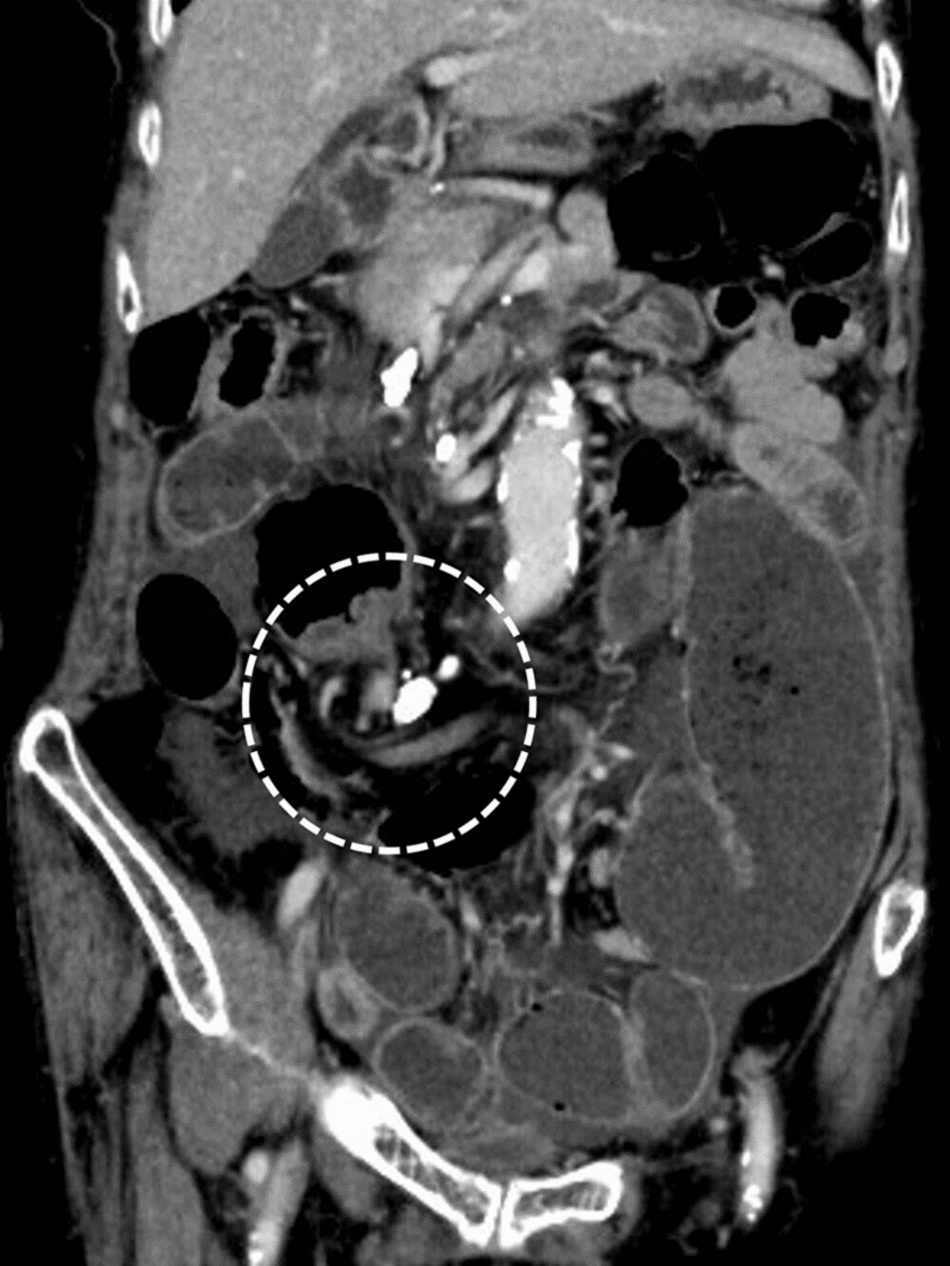
Enhanced CT of the abdomen to pelvis in case 1 The image shows a markedly dilated intestine with a whirl sign slightly to the right of the midline of the abdomen (white dashed circle).

Enhanced computed tomography (CT) of the abdomen to pelvis showed a conspicuously dilated bowel and a whirl sign slightly to the right of the midline of the abdomen (Figure 1). Based on these findings, she was diagnosed with CV, and emergency surgery was performed.

**Figure 2:**
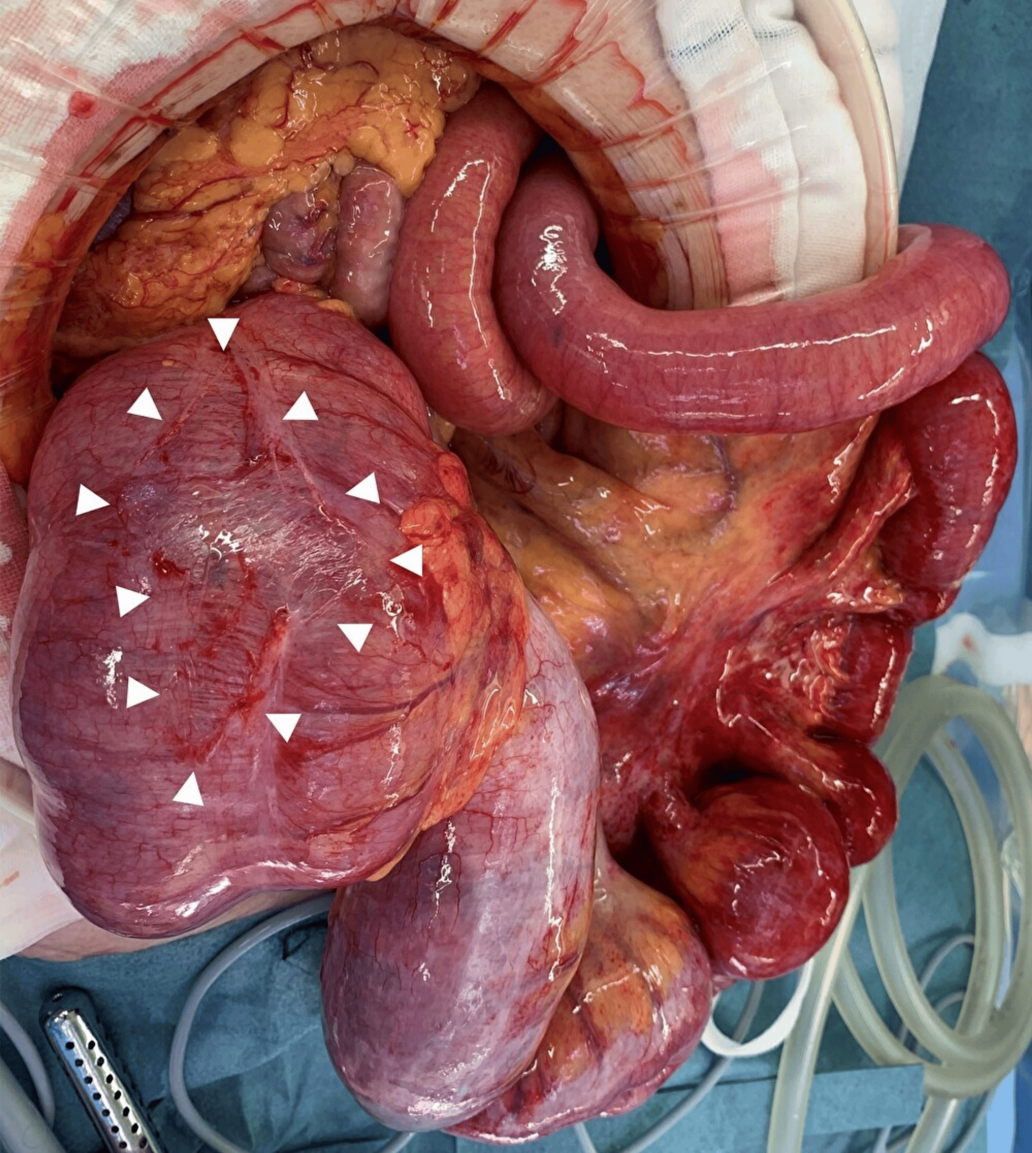
Intraoperative photograph in case 1 Photograph showing marked dilatation of the cecum and terminal ileum and serosal damage on the surface of the cecum to the ascending colon (white arrowheads).

**Figure 3:**
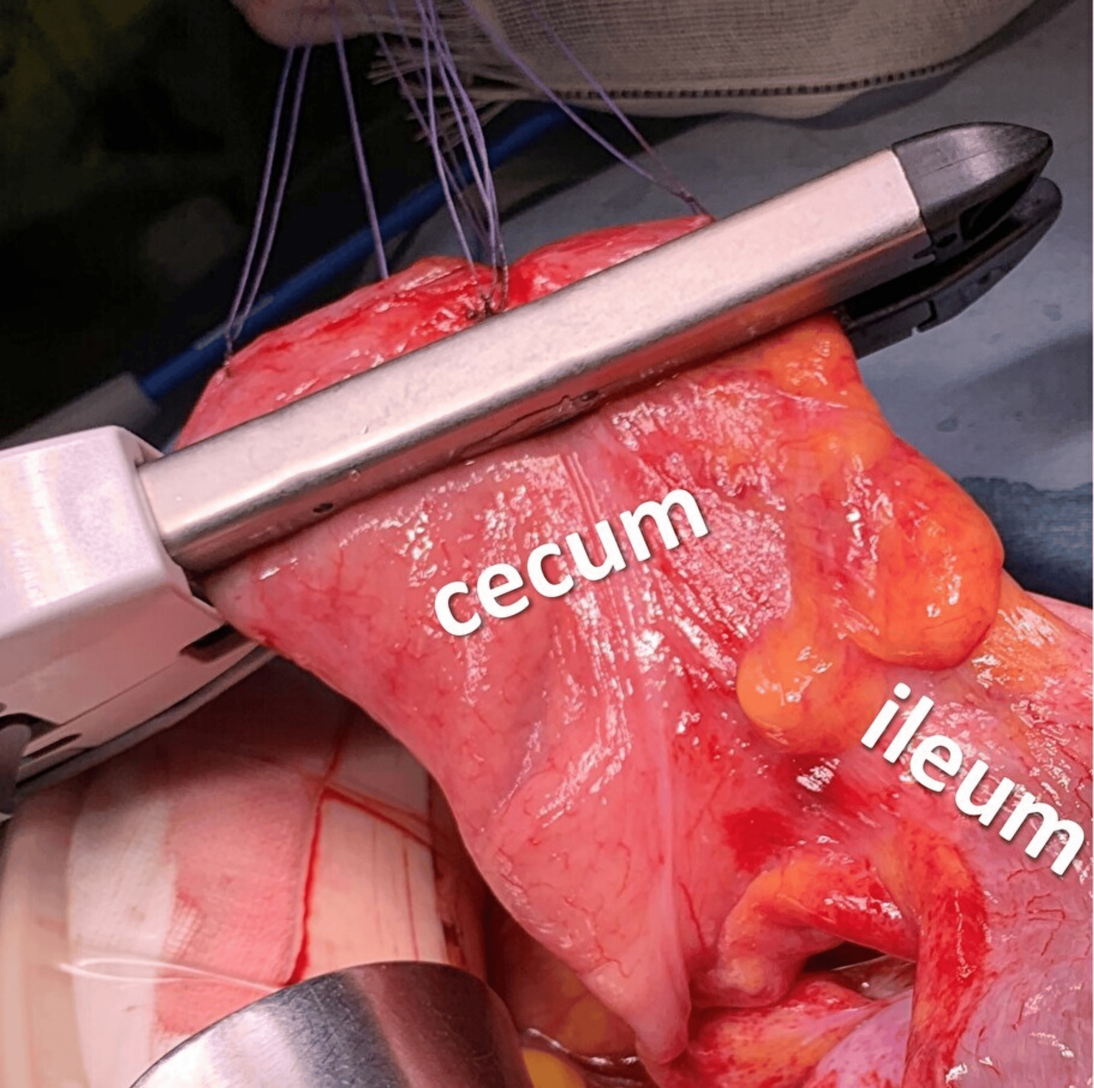
Intraoperative photograph in case 1 Photograph showing partial resection of the damaged cecum using a linear stapler.

**Figure 4:**
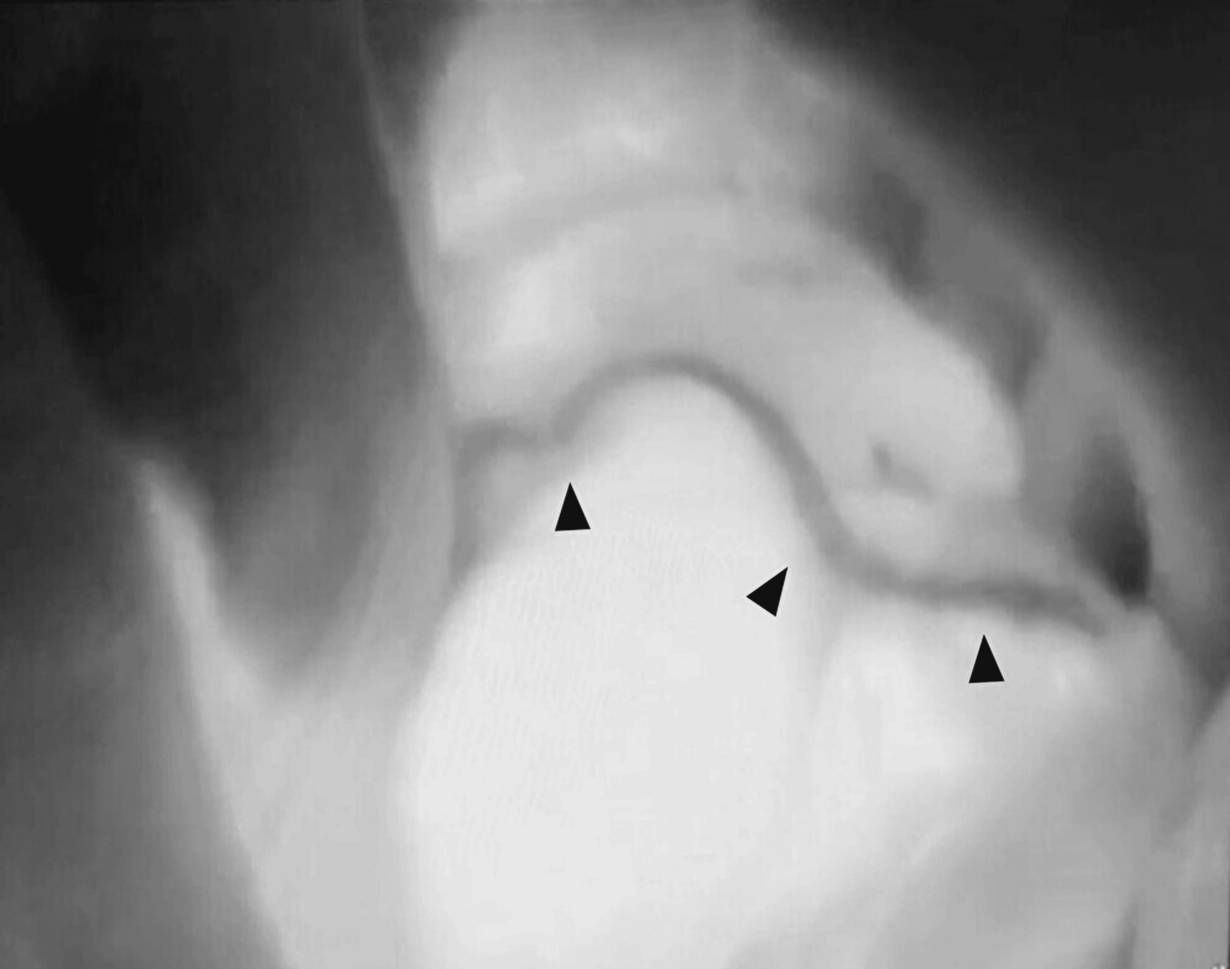
Intraoperative ICG image in case 1 Intraoperative ICG imaging showed strong fluorescence in the cecum, with the stapler curved line during partial colon resection appearing non-fluorescent (black arrowheads).

**Figure 5:**
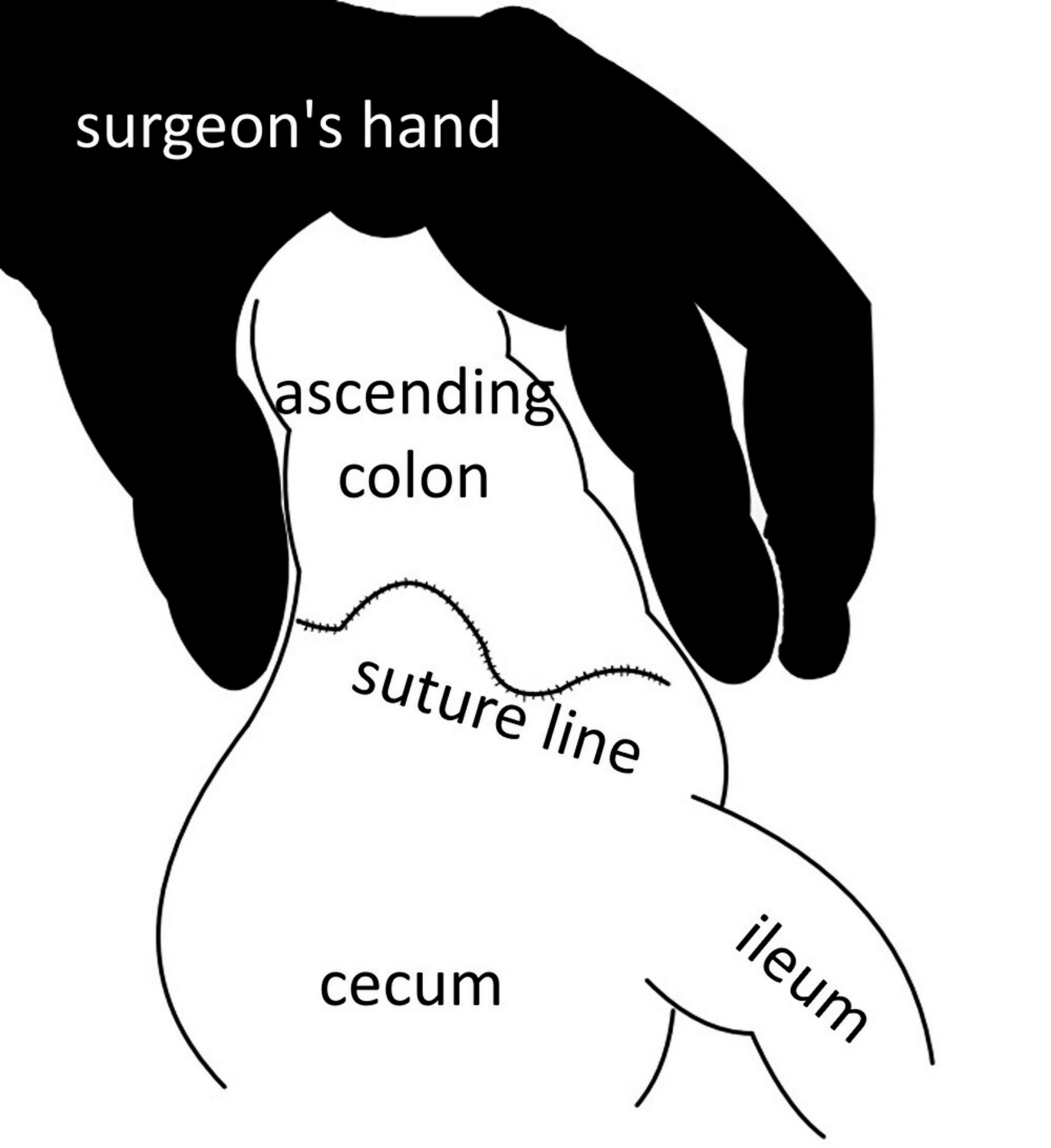
Schema of ICG image Schema showing a scene in which the surgeon's hand is grasping the colon while ICG fluorescence imaging is performed. This is an original image.

In intraoperative findings in laparotomy, a dilated cecum, ascending colon, and terminal ileum were observed. The cecum to the ascending colon was not fixed to the retroperitoneum, indicating the presence of a mobile cecum. The ileocecal region was twisted clockwise by about 270°. After careful de-rotation, detailed observation revealed serosal damage of approximately 5 cm on the surface of the cecum to the ascending colon, which was thought possibly to be due to increased intraluminal pressure (Figure 2). The site was partially resected wedge-shaped using a linear staple (Figure 3). Because the color of the ileocecal region was not clearly ischemic, ICG was used to confirm whether blood flow was sufficient. After an intravenous injection of 12.5 mg of ICG, a 20-ml saline flush was performed, and the serosa of the ileocecal region was examined using a CCD camera (pde-neo, Hamamatsu Photonics K.K., Hamamatsu, Japan). Although only the staple line of the partial colonic resection appeared as a black curve, fluorescence was immediately observed around the ileocecal region after ICG injection, confirming excellent blood flow (Figures 4-5).

**Figure 6:**
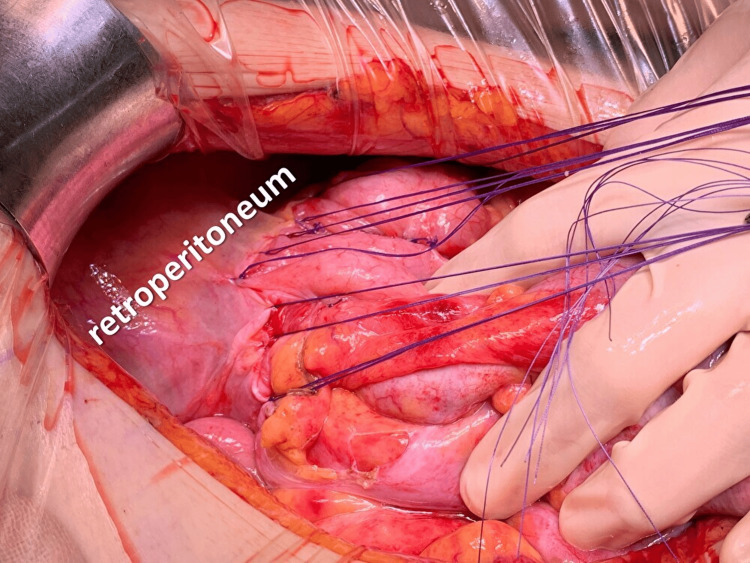
Intraoperative photograph in case 1 Intraoperative photography shows a scene in which the cecum and ascending colon are secured to the retroperitoneum using nine absorbable sutures.

The patient's serum Alb of 3.7 g/dL and stable hemodynamics indicated that ileocecal resection or right hemicolectomy with bowel anastomosis would likely be acceptable, and ileocecal resection was considered the first choice. However, based on a comprehensive assessment of the above information (that is, good intestinal blood flow, many coexisting diseases, and administration of steroids and antiplatelet drugs), the decision was made intraoperatively to perform cecopexy, which can be performed safely, rather than intestinal resection, although there was some concern about CV recurrence. The cecum and ascending colon were secured to the retroperitoneum using 3-0 absorbable sutures with nine stitches (Figure 6). The operative time was 123 min, with a blood loss of 35 ml. The postoperative course was uneventful, and the patient was discharged on postoperative day (POD) 14. At 3.5 years after surgery, there has been no recurrence of CV.

Case 2

**Figure 7:**
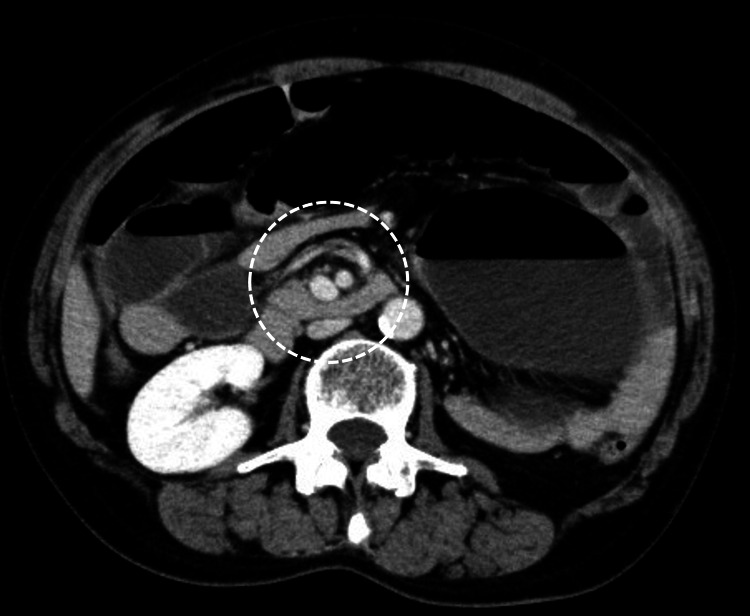
Enhanced CT of the abdomen to pelvis in case 2 The image demonstrates an extremely dilated intestine with a whirl sign slightly to the right of the midline of the abdomen (white dashed circle).

The patient was a woman in her 50s who was attending the hospital for depression (Table 1). She also had a history of surgery for left breast cancer. She came to our hospital due to abdominal distention and vomiting from the previous night. Her vital signs were BT 37.5°, HR 92 bpm, and BP 133/85 mmHg. Blood tests showed a WBC count of 9,200/µL and a CRP level of 12.11 mg/dL, indicating an elevated inflammatory response (Table 2). A CT scan of the abdomen to pelvis showed marked dilatation of the cecum and a whirl sign, leading to the diagnosis of CV (Figure 7).

**Figure 8:**
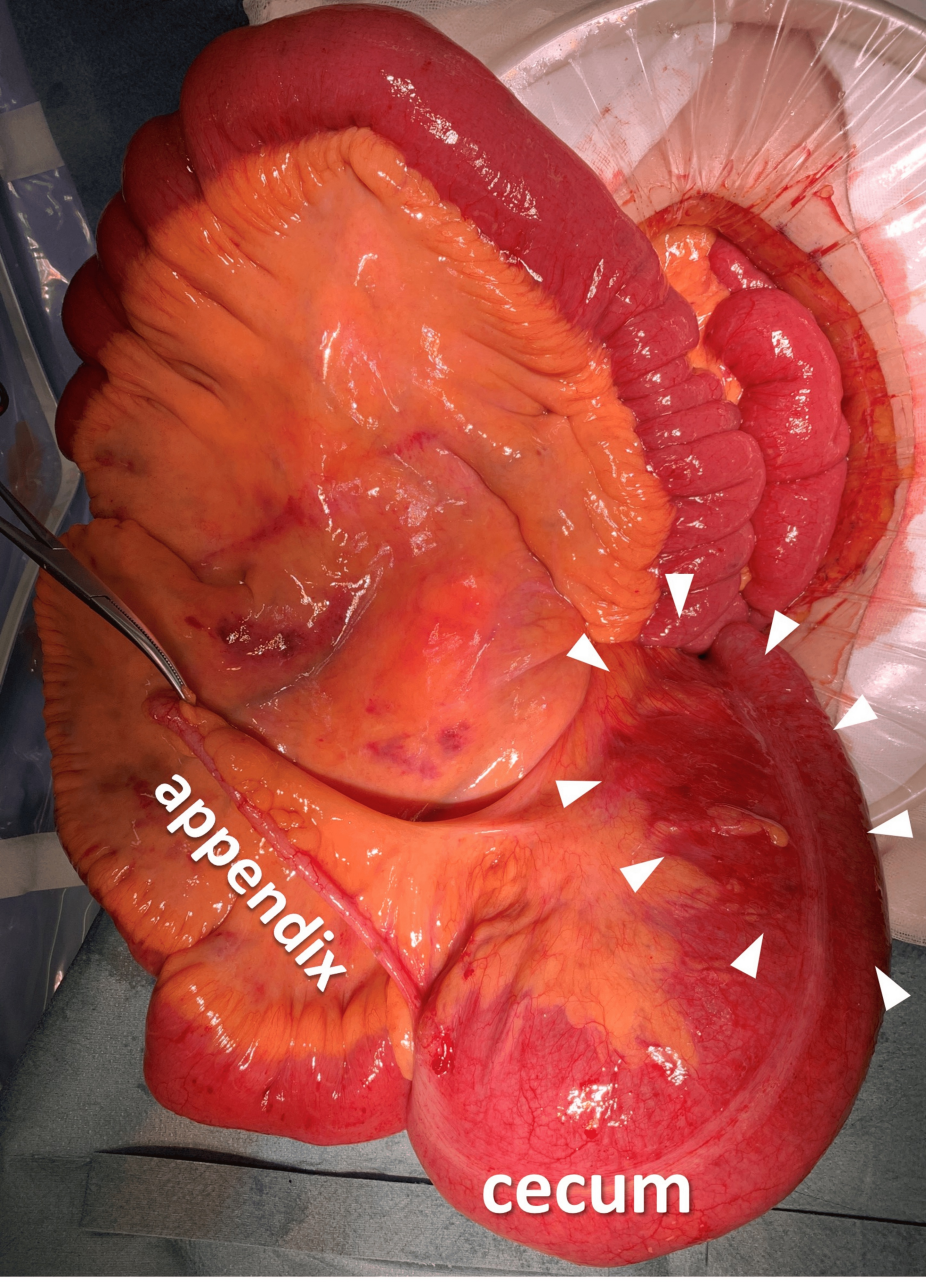
Intraoperative photography in case 2 Intraoperative photography shows markedly dilated cecum with serosal color changes, indicating ischemic changes (white arrowheads).

Emergency surgery was immediately performed. Intraoperative findings revealed that the cecum was markedly dilated and twisted 270° counterclockwise (Figure 8). We performed ileocecal resection after observing ischemic changes on the cecum's serosa.

The operative time was 126 minutes, and the blood loss was 40 ml. Postoperatively, the bowel movements were paralyzed, and a nasointestinal decompression tube was inserted, which delayed the start of oral intake, prolonged the hospital stay, and finally led to discharge on POD 41. Histopathological findings revealed acute ischemic necrosis in the cecum. Three years have passed since the surgery, and no problems have occurred with regard to the intestines.

Case 3

**Figure 9:**
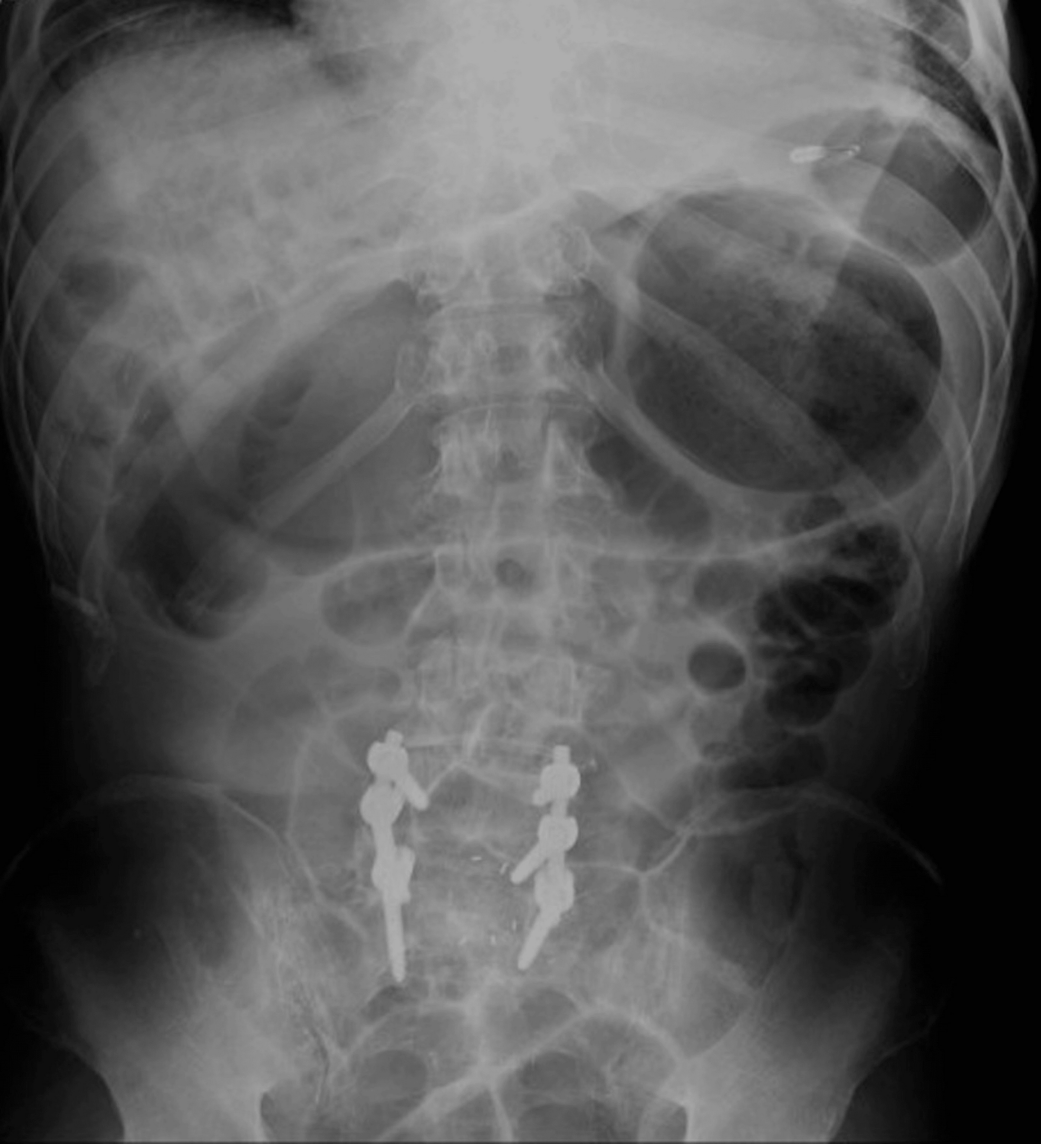
Plain abdominal X-ray in case 3 Plain abdominal X-ray shows a significant amount of intestinal gas, mainly located in the upper abdomen.

**Figure 10:**
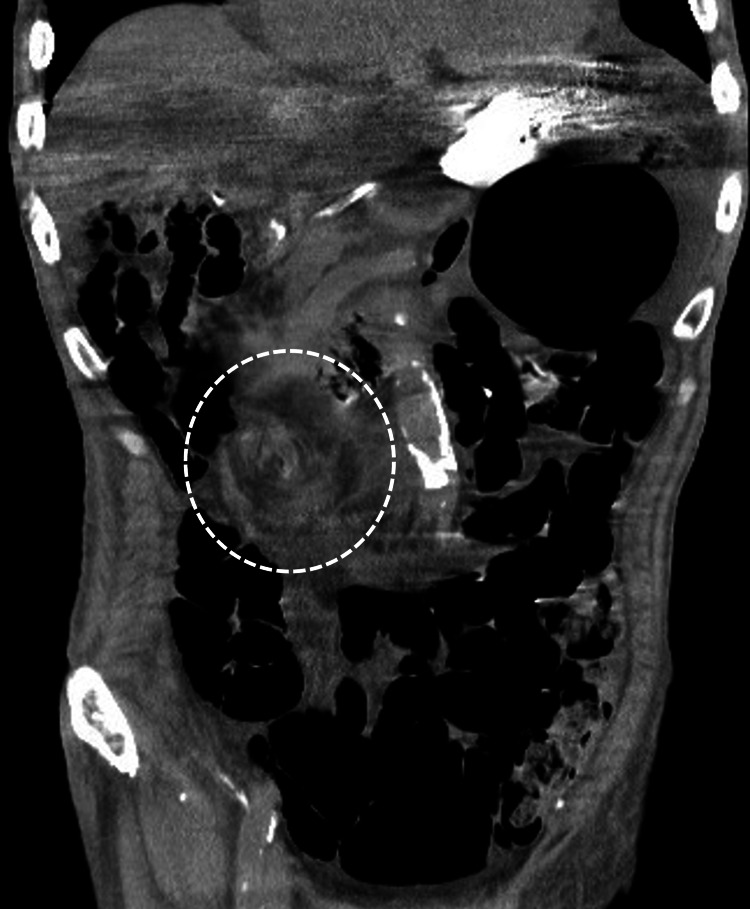
Plain CT of the abdomen to pelvis in case 3 Plain CT of the abdomen to pelvis shows a lot of intestinal gas and a suspicious whirl sign slightly to the right of the midline of the abdomen (white dashed circle).

The patient was a man in his 70s who had been attending the internal medicine department for cardiac amyloidosis and heart failure (HF) but was basically bedridden for an extended period of time (Table 1). His comorbidities included atrial fibrillation, non-sustained ventricular tachycardia, HT, DM, chronic kidney disease, and hyperuricemia. He had also undergone chemoradiotherapy for lung cancer. He visited the hospital because of abdominal pain that had lasted for a few days. On a plain abdominal X-ray, there was marked bowel dilatation in the upper abdomen, and a suspicious whirl sign was demonstrated on a plain CT scan of the abdomen to pelvis, leading to a diagnosis of CV (Figures 9-10).

**Figure 11:**
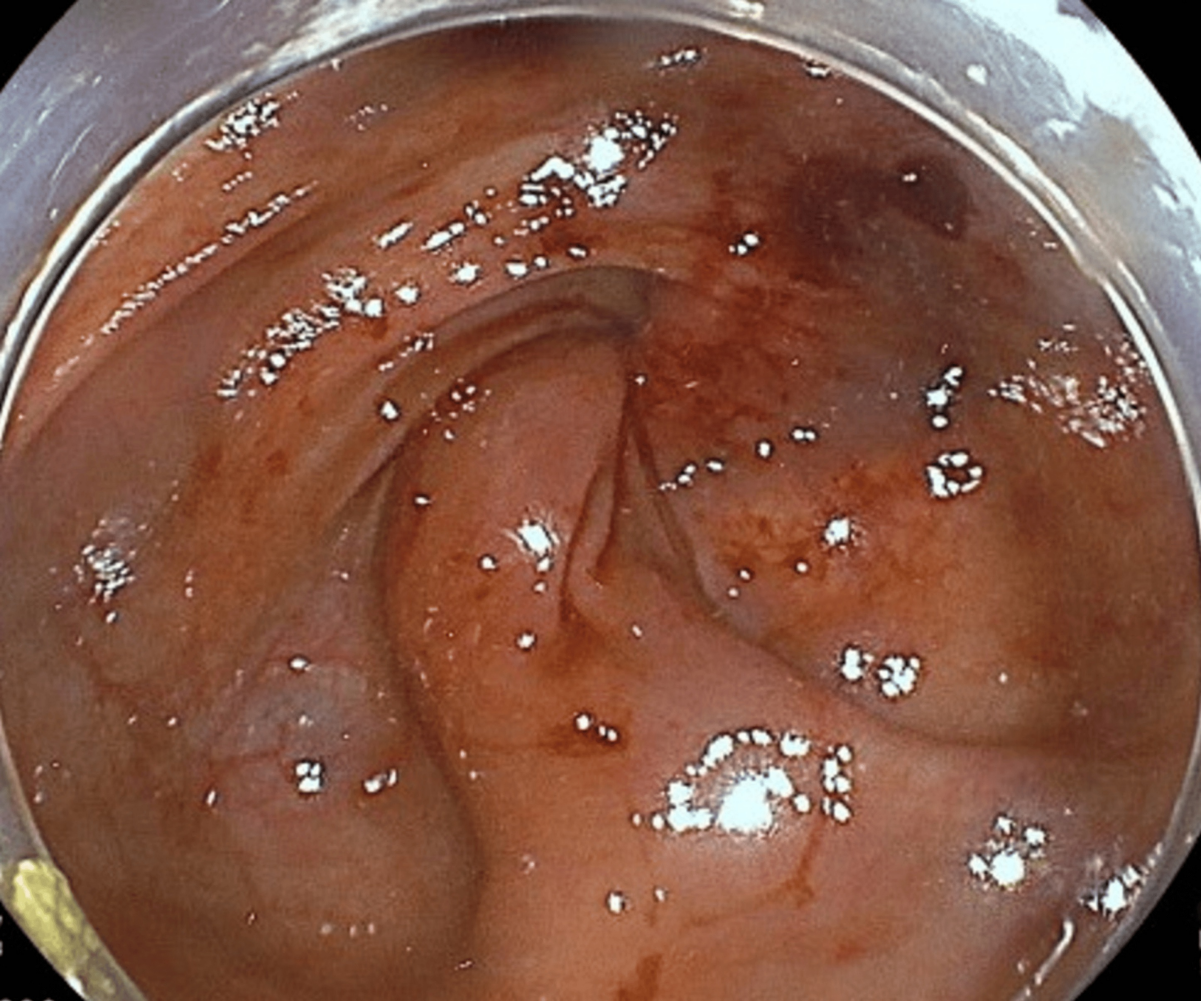
Preoperative colonoscopy in case 3 The patient had a twisted colon through which the endoscope could not pass any further toward the oral side.

The numerous comorbidities and the risk of surgery due to decreased cardiopulmonary function led us first to attempt a colonoscopic intervention. However, the colonoscope could not be passed through the torsion of the ascending colon, and the endoscopic intervention was unsuccessful (Figure 11). Furthermore, during endoscopy, bloody intestinal fluid was observed from the oral side, which indicated that the intestinal tract was strangulated, and surgery was finally performed the next day.

**Figure 12:**
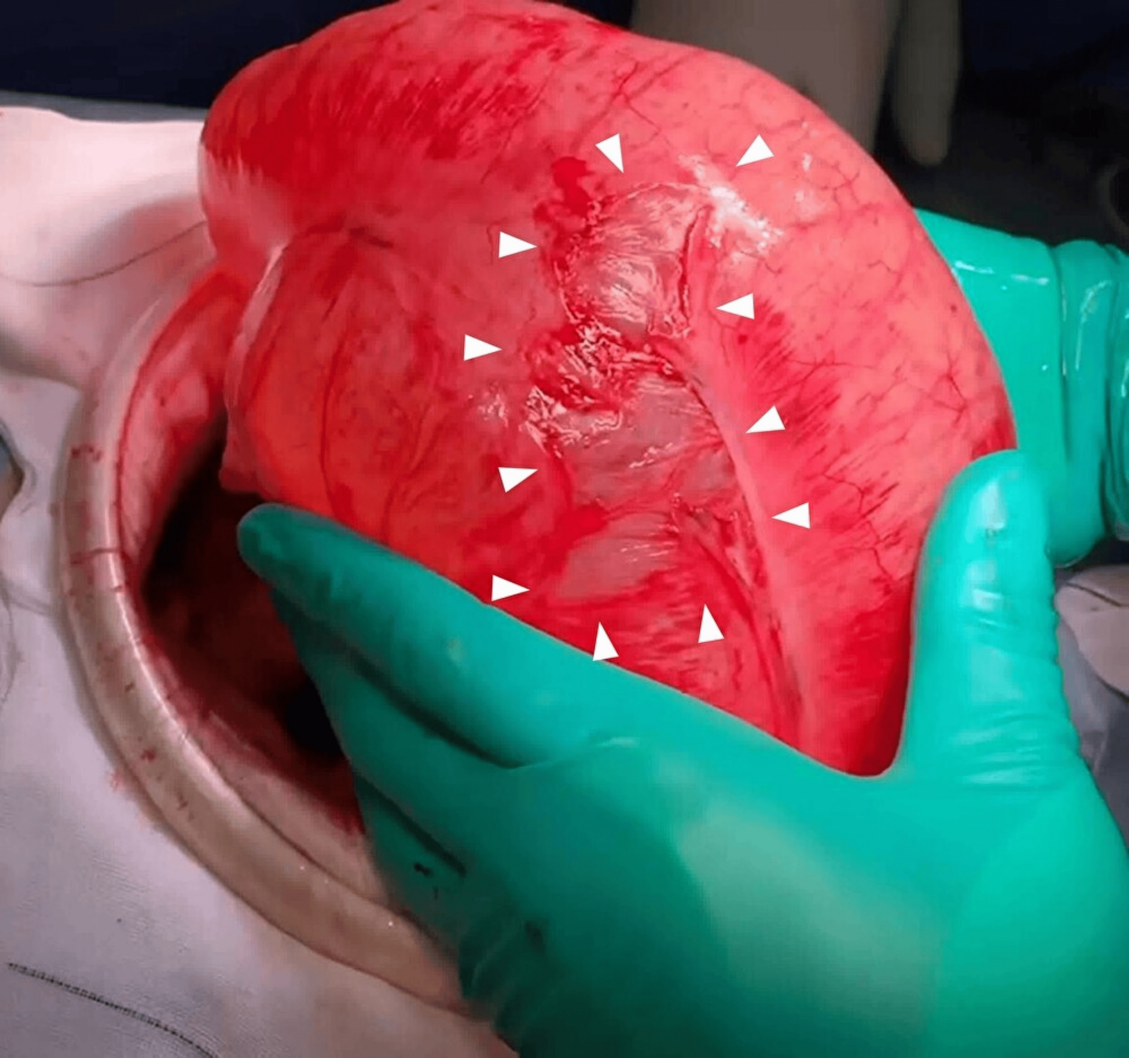
Intraoperative photograph in case 3 A dilated cecum and serosal damage (white arrowheads).

**Figure 13:**
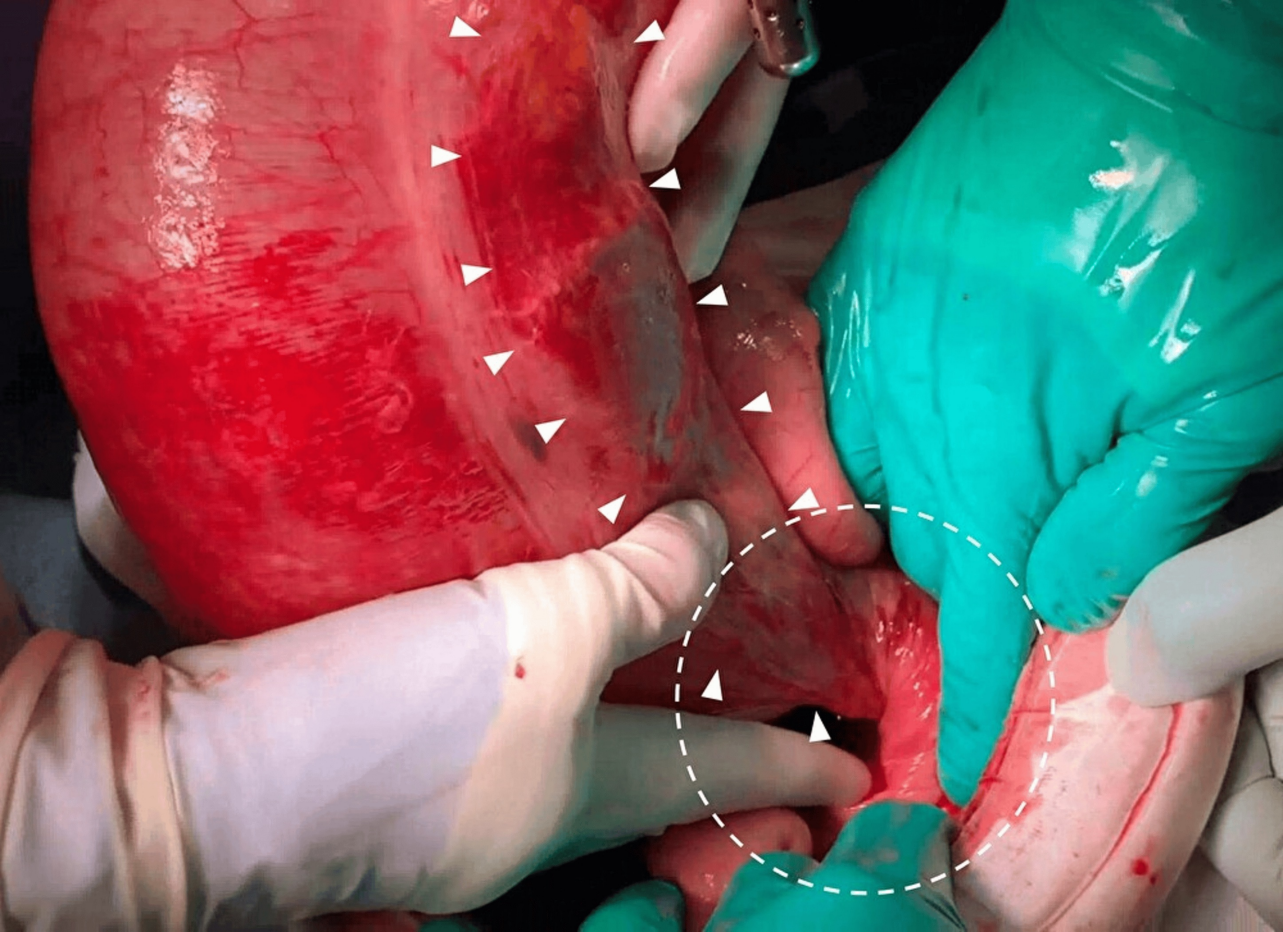
Intraoperative photograph in case 3 A detailed view of part of the twisted segment, showing the dilated cecum twisted counterclockwise by 360° (white dashed circle). The color of the serosa of the colon had changed, suggesting ischemic changes (white arrowheads).

Preoperative vital signs were relatively stable: BT 36.0°, HR 60 bpm, respiratory rate 14 breaths/min, and BP 100/60 mmHg. Intraoperative findings revealed serosal damage on the surface of the cecum, which was thought to be probably due to increased intraluminal pressure, similar to case 1 (Figure 12). The cecum was twisted 360° counterclockwise, and color changes suggestive of ischemia were observed in parts of the colonic serosa (Figure 13). Ileocecal resection was performed without ICG imaging because the patient was hemodynamically stable, and ischemic changes were suspected on preoperative and intraoperative findings. The operative time was 114 min, with a blood loss of 250 ml. Histopathology revealed a fissure of the muscularis propria, segmental oligoganglionosis, and amyloidosis in some areas. Postoperatively, anastomotic bleeding was observed twice, and hemostasis was performed by colonoscopy. On POD 69, the patient was discharged, despite the difficulty in controlling his HF and BP. Unfortunately, he died five months after the surgery, during which time no intestinal events occurred.

## Discussion

Colon volvulus is a relatively rare disease that shows regional disparities. In particular, in the region known as the “volvulus belt," which includes Africa, South America, Russia, Eastern Europe, the Middle East, India, and Brazil, 13-42% of cases of intestinal obstruction are due to colon volvulus [2]. The regional variation is thought to be due to anatomical variations, dietary habits, altitude, cultural factors, and endemic infections [2]. Although Japan is not included in the “volvulus belt,” we experienced three CV cases in a period of nine months. Regarding the types of colon volvulus, sigmoid volvulus is the most common (80%), followed by CV (15%) and transverse colon volvulus (3%) [5]. However, it is of concern that the frequency of CV has increased more rapidly than that of sigmoid volvulus in recent years, making it particularly important to establish clear information on CV [6]. Sigmoid volvulus is more common in men older than 70, while CV is more prevalent in women younger than 60. This difference occurs because the cecal mesentery may be stretched in pregnancy, and space can be created in the pelvis after hysterectomy [2].

The fundamental cause of CV is the presence of a mobile cecum, which is thought to be triggered by risk factors of constipation, high-fiber diet, laxative use, history of abdominal surgery or laparoscopic procedures, pregnancy, prior colonoscopy, stretching of the cecal mesentery, and adhesions, all of which can cause the torsion [2]. Furthermore, other than the primary diseases identified as risk factors, many patients have congenital anomalies, cerebral palsy, or mental developmental delay until their 40s, and many in their 70s or older have a history of cerebrovascular disorders, such as cerebral infarction [7]. In reviewing our cases, not all of the comorbidities match the CV risk factors listed above, but several comorbidities were present, including a history of abdominal surgery, connective tissue disorders, steroid use, psychiatric disorders, and prolonged bed rest.

The presence of a mobile cecum is clearly important in CV, but the details of this condition are not well known. The frequency of mobile cecum is estimated to be 10-20% [8], and Garude and Rao reported this finding in 18/110 cases (16.4%) in a review of surgical cases of appendicitis [9]. Given this incidence, it is clear that a relatively large number of people have the potential for CV development.

CT is effective for the diagnosis of CV, with almost 100% sensitivity and greater than 90% specificity [2]. In fact, in all three of our cases, the preoperative diagnosis using CT was accurate. Furthermore, CV is classified into three types: axial torsion type (type I), loop type (type II), and cecal bascule type (type III) [10,11]. In type I, the cecum undergoes axial torsion, rotating along its longitudinal axis; in type II, the distended cecum twists and inverts; and in type III, the distended cecum folds anteriorly without torsion. Diagnostic criteria on CT involve distinguishing features: in the CV of types I and II, a conspicuous "whirl sign" is evident, and the cecum is observed in the lower and upper abdomen, respectively, whereas type III CV is characterized by the absence of a whirl sign, with the cecum located in the central abdomen. The reported frequencies are 40% for type I, 40% for type II, and 20% for type III. This classification is crucial because types I and II are more prone to ischemic change due to intestinal torsion than type III, necessitating urgent intervention [11,12]. Thus, if a whirl sign is observed on CT, CV is more likely to be type I or II, indicating more urgency. Such a whirl sign was confirmable in all three cases reported here. Intraoperative findings showed approximately 270°, 270°, and 360° twisting, respectively, and retrospectively, all three cases were, in fact, found to be type II CV. Preoperative imaging cannot always diagnose CV, and it has been reported that 10% of cases are diagnosed intraoperatively [5].

Regarding treatment, the success rate of endoscopic intervention in sigmoid volvulus is estimated to be 70-95%, while that for CV is estimated to be 30% [2]. Therefore, as soon as a CV diagnosis is made, management with emergency surgery in mind is crucial. Approximately 61% of CV cases are associated with intestinal necrosis [2], and delayed treatment can lead to bowel ischemia, necrosis, perforation, and, ultimately, acute peritonitis [5]. In many cases, the main surgical procedure is ileocecal resection, followed by cecopexy and cecostomy [2,3]. Madiba et al. found a relatively high mortality of 17% to 32% for cases undergoing bowel resection before 1990 but a decrease of 0% to 18% after 1990 [3]. This improvement can be attributed to improved anesthesia techniques and intensive care unit management. Recurrence of CV is considered to be preventable by ileocecal resection. Mortality differs significantly in surgical procedures that do not involve bowel resection, with rates of 0% to 18% in cecopexy and 0% to 40% in cecostomy. However, the average recurrence rate after cecopexy was 16%, while recurrence after cecostomy was 0% in most reports except one [3]. Thus, cecopexy is a safer surgical procedure but is associated with a higher risk of recurrence. A comparison of bowel resection and non-bowel resection cases treated after 1990 indicates higher mortality in non-resection cases, which may be due to bias since higher-risk cases generally do not undergo bowel resection [3]. We experienced two cases of ileocecal resection and one case of cecopexy, but with no fatal complications or CV recurrence. Thus, a comprehensive consideration of safety, recurrence of CV, and the patient's overall condition appears to be important in these cases.

Recently, minimally invasive laparoscopic ileocecal resection and cecopexy have been performed for CV [1,4,6]. In the United States, laparoscopic bowel resection for colon volvulus in young patients with few comorbidities has increasingly been used since around 2010, and mortality is lower for laparoscopic surgery than for open surgery [6]. However, Bauman and Evans do not recommend laparoscopic surgery for CV because, unlike sigmoid volvulus, there is significant dilatation of the volvulized loop and proximal intestine [2]. Given such considerations, laparoscopy for CV is still considered to be controversial. Although our team is well accustomed to minimally invasive surgery such as laparoscopy and robotic surgery, all three of the cases reported here had significant intestinal dilatation. Therefore, laparotomy was performed, and good outcomes were achieved. The future introduction of minimally invasive surgery into the CV procedure will depend on the severity of intestinal dilatation, whether pneumoperitoneum is possible, and the patient's general condition.

In this study, we were able to use ICG fluorescence imaging to evaluate whether intestinal blood flow was sufficient in case 1 and then perform cecopexy. In cases 2 and 3, pre- and intraoperative findings indicated a high possibility of intestinal ischemia, and the decision was made to perform ileocecal resection immediately, so blood flow evaluation using ICG was not performed. Although ICG imaging may not be performed in some facilities, we routinely use ICG fluorescence during colorectal cancer surgeries, and our surgeons, anesthesiologists, and nurses are familiar with the use of ICG [13]. This allowed the performance of ICG imaging as usual in this case, despite the emergency surgery situation, and the patient benefited as a result. If the patient's overall condition is poor and the intestinal blood flow assessment using ICG is also poor, it may be necessary to consider intestinal resection without anastomosis and the creation of a stoma. We were unable to find any other reports of cecopexy with ICG imaging in emergency surgery for CV, so this report is considered novel from this perspective. Although ileocecal resection is still the first choice [3], our case also suggests that the availability of ICG imaging can increase the possibility of choosing safer cecopexy over bowel resection for patients with CV who have a poor general condition.

## Conclusions

Emergency cecopexy, ileocecal resection, and colonoscopy were all performed in three CV cases with good outcomes. CT was effective for diagnosis in each case, and cecopexy was safely performed after ICG imaging for the patient at very high risk and with a low possibility of intestinal ischemia. When selecting a surgical procedure, although the golden standard is ileocecal resection, it is important to make a comprehensive judgment, including safety. We believe that these cases provide good evidence for appropriate approaches to the diagnosis and treatment of CV that will be of value in the management of future cases.
